# Severe Pelvic Obliquity Affects Femoral Offset in Patients with Total Hip Arthroplasty but Not Leg-Length Inequality

**DOI:** 10.1371/journal.pone.0144863

**Published:** 2015-12-16

**Authors:** Xiaoxiao Zhou, Qi Wang, Xianlong Zhang, Yunsu Chen, Xiaochun Peng, Yuanqing Mao, Yang Yang, Beigang Fu, Xiuhui Wang, Tingting Tang

**Affiliations:** 1 Department of Orthopedics, Zhoupu Hospital of Pudong District, Shanghai University of Medicine & Health Sciences, Shanghai, China; 2 Department of Orthopaedics, Shanghai Sixth People's Hospital, Shanghai JiaoTong University, Shanghai, China; 3 Department of Orthopaedics, Ninth People's Hospital, Shanghai Jiao Tong University, Shanghai, China; 4 Wenzhou Medical University, Wenzhou, Zhejiang, China; Louisiana State University, UNITED STATES

## Abstract

Leg-length inequality is an extensively studied complication of total hip arthroplasty in normal patients. However, few studies have focused on the pelvic obliquity of coronal pelvic malrotation. We hypothesized that pelvic obliquity with a fixed abduction/adduction contracture deformity of the hip may intraoperatively affect the release of soft tissues, ultimately resulting in a leg-length inequality. This study also investigated whether the femoral and vertical offsets of total hip arthroplasty were correlated with pelvic obliquity. This prospective study divided 98 patients into six groups based on the inclination of pelvic obliquity before total hip arthroplasty. Leg-length inequality, variation of pelvic obliquity, offset, and vertical offset were measured after total hip arthroplasty. Leg-length inequality and vertical offset were not significantly different among groups, whereas the variation of pelvic obliquity was significantly higher in type IIC pelvic obliquity than in other groups. Type IC pelvic obliquity had a significantly shorter offset than did the other groups, which may have been an important factor leading to type IC pelvic obliquity. Pelvic obliquity exhibited no significant effect on leg-length inequality in patients with total hip arthroplasty. A shorter offset may be caused by the higher tension of the abductor in the operated hip, which may result in the formation of type IC pelvic obliquity. Releasing the abductor contracture and restoring femoral offset are important for increasing hip stability and maintaining pelvic balance following total hip arthroplasty.

## Introduction

Total hip arthroplasty (THA) can relieve hip pain [1], correct hip deformities, provide hip stability [2], restore hip function [3], and improve a patient’s quality of life [4]. Leg-length inequality (LLI) is a common complication after THA, which may also cause subjective problems for patients. This complication is often associated with limping, low back pain, greater trochanteric bursitis, lumbar scoliosis, and degenerative hip disease [5, 6]. LLI following THA is a definite source of patient complaints. Some patients who are dissatisfied with LLI, particularly with the increases in leg length, commonly wear a contralateral shoe lift. Significant lengthening of the leg can also lead to several complications, such as nerve palsy, specifically sciatic nerve palsy. As a result of patient dissatisfaction, LLI following THA is a leading cause of litigation.

Pelvic obliquity (PO), which here refers to infrapelvic obliquity, is a coronal obliquity of pelvic malrotation that is commonly secondary to abduction or adduction hip contractures and is frequently related to THA [7, 8]. A previous study found that PO could result in the malpositioning of the acetabular component and may diminish the longevity of endoprostheses in THA [8].

The sensation of LLI after THA has been described in normal patients [9], but only one study on functional limb-length inequality (FLLI) after THA with respect to PO has been reported. However, considering that that study was retrospectively reviewed, the PO angles were not fundamentally measured [10]. Thus, the actual effects of PO on LLI in patients with THA remain unknown. Further studies are necessary to clarify these effects.

**Fig 1:**
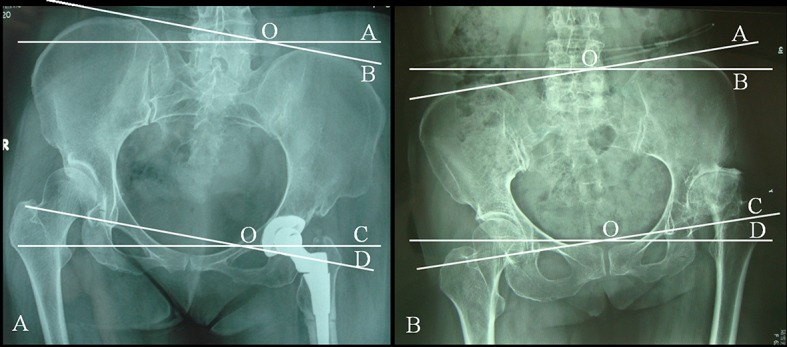
Anteroposterior image of a patient illustrating how the degree of PO is measured. A. A patient with type IC pelvic obliquity after total hip arthroplasty; the iliolumbar angle was 8.37°. B. A patient with type IIC pelvic obliquity before total hip arthroplasty; the iliolumbar angle was 7.79°. One line was drawn on the anteroposterior radiographs of the pelvis and lower lumbar spine, and this line connected the apices of both iliac crests (OA) and another along the bottom of the fourth lumbar vertebra (OB). The iliolumbar angle was measured at the convergence of these two lines or at the angle of the trans-teardrop (OC) and horizontal lines (OD) when the lower lumbar vertebra could not be completely viewed in the anteroposterior pelvic radiographs.

Some intraoperative factors may affect LLI after THA, including anesthesia type [11]. Theoretically, the type I PO deformity is characterized by an abduction contracture deformity in the affected hip, as well as a pelvis that is inclined forward to the affected side and lower on the side of the shorter leg (Fig 1A). Conversely, the type II deformity is characterized by an adduction contracture deformity in the affected hip and a higher pelvis that is inclined forward to the healthy side (Fig 1B) [12]. We hypothesize that PO with a fixed abduction/adduction contracture deformity of the hip may intraoperatively affect the release of soft tissues, ultimately resulting in LLI. Thus, a prospective clinical study was conducted to test this hypothesis.

Although the main limitations of the hip joint are attributed to the soft tissue structures around the hip, the importance of the bony architecture has recently become of greater interest [13]. Femoral offset is correlated with hip stability [14], which Charnley [15] considered to be a factor under the control of the surgeon during THA. Occurrences of LLI can be attributed to the absence of lateralized neck options, which necessitates the increase of the vertical offset to improve overall hip stability [16]. Considering that femoral and vertical offsets may play a role in maintaining hip and pelvic balance, this study also investigated whether the femoral and vertical offsets of THA were correlated with PO.

## Material and Methods

A total of 183 consecutive patients who underwent THA Shanghai Sixth People's Hospital Affiliated with Shanghai JiaoTong University between April 2013 and June 2013 were considered for this study. This experiment was approved by both the institutional research ethics and scientific committees of the hospital, and written informed consent was provided by participants for their clinical records to be used in this study. Eighty-five patients who experienced femoral neck fracture or bilateral one-stage THA that did not reflect the reality of PO were excluded. Thus, 98 patients (35 men and 63 women) were ultimately included. The preoperative diagnoses of these patients were as follows: developmental dysplasia of the hip (35 cases), avascular osteonecrosis of the femoral head (39 cases), ankylosing spondylitis (3 cases), septic arthritis (1 case), primary osteoarthritis (3 cases), and secondary osteoarthritis (17 cases). All patients had non-cemented acetabular and femoral components (THA components were provided by Stryker, Biomet, Wright, LINK, and Depuy). Ninety-six patients had ceramic-on-ceramic bearing surfaces, and two had metal-on-polyethylene (PE) bearing surfaces. Patient ages ranged from 20 to 82 years (58.02 ± 10.47 years). Preoperative and postoperative radiographs from all patients were reviewed.

### Operative Procedures

THA was performed as described in a previous report [8]; that is, a posterolateral approach to the hip was used while patients were in the lateral decubitus position. The neck length and size of the prostheses were selected based on the correct LLI that matched the preoperative templating and gluteal muscle tension. Selective release for the contracture of the abductor or adductor was performed if joint reduction was difficult, flexion contracture remained after joint reduction, or insufficient external rotation was achieved.

### Radiological Classification

Standing preoperative and postoperative anteroposterior (AP) pelvic and hip radiographs were taken for each patient two days before and two days after the operation during their hospital stay. These radiographs were used as parameters. Both legs were internally rotated 15° to obtain the pelvic radiographs [17]. The authors assessed PO in relation to the hips and legs by reviewing these radiographs. PO was classified into two major deformity types: I (Fig 1A) and II (Fig 1B). The direction of pelvic inclination was used relative to the short leg as the basis for classification, with modifications based on the purpose of the present and previous studies [8, 12], i.e. based on the inclination of preoperative and postoperative PO, each deformity type was further classified into six subtypes depending on the severity of the PO angle of inclination: 0°–3° (types IA and IIA), 3°–6° (types IB and IIB), and >6° (types IC and IIC) [8]. The patients were then divided into six groups, namely, 0°–3° (group types IA and IIA), 3°–6° (group types IB and IIB), and >6° (group types IC and IIC) groups.

### Radiological Measurements

#### PO inclination

PO angles were measured as described in previous studies, with modifications (Fig 1A and 1B) [8, 12]. One line was drawn on the AP radiograph of the pelvis with respect to the lower lumbar spine; this line connected the apices of both iliac crests, and another line was drawn along the bottom of the fourth lumbar vertebra [8]. The iliolumbar angle was measured at the convergence of these two lines or at the angle of the trans-teardrop and horizontal lines when the lower lumbar vertebra could not be completely viewed on the AP pelvic radiographs. Postoperative variation of PO (vPO) was also measured and calculated. Negative vPO values indicated that the preoperatively classified PO converted to another type postoperatively.

#### Offset

**Fig 2:**
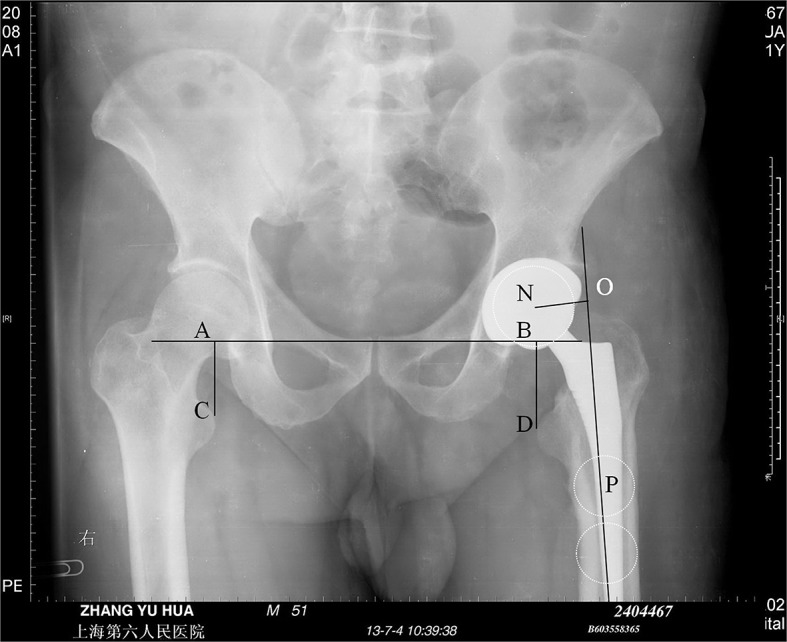
Schematic indicating the method for measuring relevant parameters from anteroposterior pelvic and hip radiographs. Point N is the center of the femoral head, line OP is the anatomic axis of the femur, NO is perpendicular to line OP, and the distance of line NO is the hip offset. Points C and D are the most prominent points of the lesser trochanter, line AB is the trans-teardrop line with lines CA and DB drawn perpendicular to line AB, and the distances of lines CA and DB are femoral vertical offsets. The discrepancy between the lengths of lines DB and CA was documented as the LLI.

Offset is the perpendicular distance between the long axis of the femur and the center of rotation of the femoral head [18] (Fig 2).

#### Vertical offset

Vertical offset is the distance between the trans-teardrop line and the medial apex of the lesser trochanter [18] (Fig 2).

**LLI.** LLI is the length of a line perpendicular and connecting to the trans-teardrop line and the most prominent point of the lesser trochanter on AP pelvic and hip radiographs [19]. The discrepancy between the lengths of the vertical offset for the replaced and contralateral hip was documented as the LLI (Fig 2).

The defined anatomical landmarks included the ischial tuberosities, apex (i.e., most medial point) of the lesser trochanter, apex of the teardrop, and longitudinal axis of the femur. The teardrop was selected as a standard reference instead of the ischial tuberosity because the teardrop is a more consistent landmark [20]. The diameter of the acetabular cup on the radiograph was measured and compared with that of the actual cup to compensate for film magnification in the calculation of LLI, offsets, and vertical offsets for each patient. This step was important because of the differences in magnification based on patient size (such as less magnification in thin patients [14%] and more magnification in obese patients [26%]) [18]. Based on these measurements, differences in vertical offsets, offsets, and LLI in the operated and contralateral limbs were calculated from the preoperative and postoperative radiographs. All final absolute values were used for statistical analysis. All distances were measured in millimeters. The angles of PO inclination and the distances of offset and LLI were measured using Image-Pro Plus (Media Cybernetics, Inc. USA).

### Statistical Methods

ANOVA was performed using SPSS statistical software (SPSS 11.5; SPSS Inc., Chicago, IL). The Student-Newman-Keuls multiple comparisons test was used when multiple groups were compared. If equal variances were not assumed, Dunnett’s T3 test was used to compare differences between groups. Differences for all analyses were considered significant at *p* < 0.05. Data were presented as means ± standard deviation (SD).

## Results

The average actual diameter of the cup that was applied intraoperatively was 50.04 mm (44.00–60.00 mm). The average magnification for the measured value was 13.73% (3.79%–22.65%).

As shown in Table 1 (see also S1 Text), there were no significant differences in the preoperative PO and LLI among individuals in group IA (or IIA), IB (or IIB), and IC (or IIC) (*p* = 0.863) after THA. The leg-length measurement demonstrated that the average LLI was 6.55 ± 5.49 mm (0.24–25.80 mm). Leg shortening and lengthening were observed in 31.63% (31/98) and 68.37% (67/98) of patients, respectively. LLI in three patients (3.06%, 3/98) reached or exceeded 20 mm, and only one of these three patients exhibited a shortened leg (25.80 mm). The LLIs were within 0–5 mm in 45.92% (45/98), 0–7 mm in 66.33% (65/98), and 0–20 mm in 96.94% (95/98) of the cases. No significant differences were observed among the groups for operated vertical offset (ov-offset) (*p* = 0.359), contralateral vertical offset (cv-offset) (*p* = 0.967), operated offset (o-offset) (*p* = 0.501), or contralateral offset (c-offset) (*p* = 0.502) before THA. Meanwhile, vPO was significantly different among the groups (*p* = 0.001). The vPO for type IIC was significantly increased compared with types IA (*p* = 0.001), IB (*p* < 0.001), IC (*p* = 0.021), and IIA (*p* = 0.003). An increasing tendency was also observed for type IIC compared with type IIB (*p* = 0.196). Group type IIC showed the biggest vPO value among all of the groups.

**Table 1:** Parameters based on preoperative pelvic obliquity.

	Type IA	Type IB	Type IC	Type IIA	Type IIB	Type IIC	*p*
**Total patients (%)**	25 (25.51%)	28 (28.57%)	13 (13.27%)	18 (18.37%)	7 (7.14%)	7 (7.14%)	
**LLI**	5.96 ± 3.67	7.39 ± 7.11	7.91 ± 7.52	6.98 ± 5.32	6.24 ± 6.60	4.82 ± 3.90	0.836
**ov-offset (mm)**	35.44 ± 6.33	30.12 ± 9.09	29.07 ± 9.36	33.71 ± 11.61	31.23 ± 8.79	30.39 ± 7.74	0.421
**cv-offset (mm)**	30.52 ± 6.81	29.08 ± 8.59	27.28 ± 8.74	29.30 ± 12.06	29.19 ± 9.02	27.96 ± 4.01	0.967
**o-offset (mm)**	28.89 ± 8.27	27.63 ± 7.03	23.69 ± 8.08	28.12 ± 8.35	29.38 ± 6.77	31.72 ± 6.99	0.501
**c-offset (mm)**	25.50 ± 7.96	23.80 ± 10.78	28.99 ± 12.06	24.75 ± 7.20	28.75 ± 7.70	26.39 ± 8.04	0.502
**vPO**	2.85 ± 2.39^1^	2.21 ± 2.01^2^	3.62 ± 4.85^3^	3.58 ± 3.21^4^	5.62 ± 2.93^5^	7.34 ± 3.04	0.001

All values are expressed as the mean ± standard deviation unless otherwise noted. Differences were considered significant at

p < 0.05.

^**1**^p = 0.001

^**2**^p < 0.001

^**3**^p = 0.021

^**4**^p = 0.003

^**5**^p = 0.196.

LLI = leg-length inequality

ov-offset = operated vertical offset

cv-offset = contralateral vertical offset

o-offset = operated offset

c-offset = contralateral offset, and

vPO = variation of pelvic obliquity.

As shown in Table 2 (see also S2 Text), based on the postoperative PO, there was no significant different in LLI between types I and II PO (*p* = 0.633) after THA; importantly, no case of type IIC presented after THA. There were no significant group differences for the ov-offset (*p* = 0.421), cv-offset (*p* = 0.873), and c-offset (*p* = 0.535) after THA. However, significant differences were observed among the groups for the o-offset (*p* = 0.028). The o-offset for type IC was significantly shorter than those for types IA (*p* = 0.016), IIA (*p* = 0.004), and IIB (*p* = 0.011), and a decreasing trend was observed compared with type IB (*p* = 0.055). Group type IC showed the smallest o-offset value among all of the groups.

**Table 2:** Parameters for pelvic obliquity after total hip arthroplasty.

	Type IA	Type IB	Type IC	Type IIA	Type IIB	*p*
**Total patients (%)**	37 (37.76%)	24 (24.49%)	14 (14.29%)	17 (17.35%)	6 (6.12%)	
**LLI (mm)**	7.45 ± 6.14	5.37 ± 4.93	7.20 ± 5.92	5.90 ± 3.29	6.05 ± 7.70	0.633
**ov-offset (mm)**	33.70 ± 10.12	32.98 ± 8.06	28.32 ± 7.74	33.83 ± 9.39	31.65 ± 10.53	0.421
**cv-offset (mm)**	29.73 ± 9.87	29.03 ± 8.05	28.18 ± 7.20	30.73 ± 9.79	26.65 ± 11.22	0.873
**c-offset (mm)**	25.08 ± 8.47	24.43 ± 9.15	28.07 ± 9.43	28.07 ± 6.83	26.76 ± 7.19	0.535
**o-offset (mm)**	28.86 ± 8.51^1^	27.95 ± 6.68^2^	23.07 ± 8.96	31.13 ± 4.92^3^	32.49 ± 5.46^4^	0.028

All values are expressed as the mean ± standard deviation unless otherwise noted. Differences were considered significant at

p < 0.05.

^**1**^p = 0.015

^**2**^p = 0.055

^**3**^p = 0.004

^**4**^p = 0.011.

LLI = leg-length inequality

ov-offset = operated vertical offset

cv-offset = contralateral vertical offset

o-offset = operated offset

c-offset = contralateral offset.

## Discussion

LLI between types I and II PO were not significantly different after THA, suggesting that LLI in patients with THA was not affected by soft tissue tightness or PO. Our results are consistent with those of previous studies [10]. Only 3.06% (3/98) of the LLI reached or exceeded 20 mm, which is the clinically unacceptable threshold for THA [10, 21]. Leg length is considered corrected if the other leg is within 5 mm [22]. A slight increase in the actual leg length (i.e., by 5 mm) may produce a significant FLLI if the soft tissue structures remain tight [10]. Based on this criterion, the results showed that 45.92% (45/98) and 66.33% (65/98) of the cases exhibited an LLI within 5 and 7 mm, respectively, which agrees with previous results, wherein the leg is lengthened by 0–7 mm in 61% of cases [10].

The vPO slightly changed in patients with type I PO compared with that in those with type IIC PO. Type IIC PO showed greater variation after THA than did the other types of PO. This result implies that type IIC PO was intraoperatively easier to correct than was type I PO. The deformity of type I PO, especially type IC, was relatively irreversible and fixed. This result was indirectly demonstrated in a previous study, where type IC PO (>6°) was not easily changed intraoperatively [8].

Type IIC PO was not found after THA. This result was likely not due to an insufficient sample but rather because this type is easy to correct intraoperatively, as shown by the highest vPO values for type IIC PO.

Meanwhile, the o-offset of type IC was prominently shortened compared with that of types IA, IB, IIA, and IIB based on their postoperative PO (Table 2). However, the preoperative o-offset was not significantly different among the groups (Table 1).

**Fig 3:**
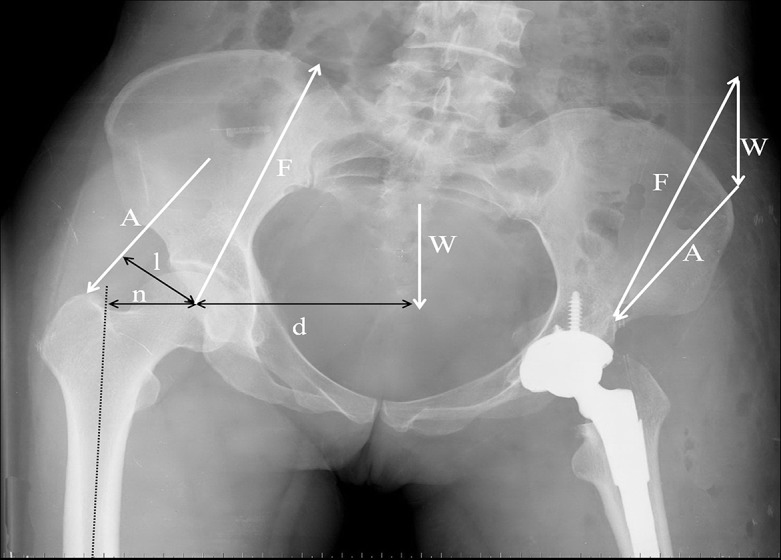
Radiograph showing the forces acting on the hip joint during a single-leg stance under equilibrium conditions. Gravitational force W, hip joint reaction force F, abductor force A, femoral shaft axial line S, abductor muscle moment arm l, force of gravity moment arm d, and femoral offset n.

As the main connection between the lower limbs and the trunk, the hip joint and its motion between these the parts of body allows for bipedalism. Enormous effort has been expended over the years to define the various forces that contribute to the joint reaction force of the human hip [13]. The free-body diagram of the forces across the hip joint under static conditions [23,24] provides an easy-to-understand basis for demonstrating the importance of subtle changes in body position or hip anatomy. Based on the free-body diagram, body position or hip anatomy allows changes in forces that can occur around the hip joint. The following forces act on the pelvis and hip joint to maintain the pelvis’s level: (1) gravitational force, W, which is the weight of the body minus the weight of the contralateral lower limb; (2) A, which is the force of the abductor muscles that maintains the pelvis level; and (3) F, which is the force exerted by the femoral head on the acetabulum, or the joint reaction force (Fig 3) [13, 24]. The sum of the force vectors A, F, and W in equilibrium is zero [23]. We hypothesized its potential mechanism, as described below; the magnitude of the joint reaction force on the femoral head is inversely proportional to the ratio of the abductor muscle’s force lever arm to the gravitational force lever arm; i.e., this ratio decreases with increasing joint reaction force. Given the gravitational force, the gravitational force lever arm remained unchanged if the abductor muscle force lever arm decreased; this phenomenon results in the increase of the force that must be generated by the abductor musculature to counteract the force of gravity on the pelvis [13, 23].

To examine the biomechanical principles involved in the function of the human hip, the normal anatomy of the proximal femur and pelvis should be considered. The muscles, ligaments, and bony structures all contribute to the equilibrium of forces that allow for controlled motion at the femoral–acetabular articulation. The significance of the local anatomy surrounding the hip should be scrutinized because of its important contribution to hip biomechanics [13]. There are difficulties associated with quantifying the effects of subtle differences in the proximal femoral and acetabular morphologies on hip biomechanics, especially because these differences have yet to be well described, such as the femoral offset. Soft tissue balance of the hip during THA is performed to restore the offset and leg length in the reconstructed hip. Reproducing a reconstructed femoral offset to within 5 mm of the native femoral offset is associated with decreased conventional PE wear [25]. The offset in THA correlates with abductor muscle function and wear, and impingement hip offset reconstruction directly relates to the position of the hip’s center of rotation [26]. A more lateral position of the femur with a greater offset increases the range of motion and decreases the incidence of impingement of the femur on the pelvis. An increase in the femoral offset and, consequently, of the lever arm of the abductor muscles theoretically increases the mechanical advantage and strength of the abductors. Previous reports suggested that the greater femoral offset after THA allows for an increased range of abduction and greater abductor strength [27]. Several conditions predispose toward abnormalities in proximal femoral or acetabular morphology [13], such as type IC with the shorter femoral offset, after THA. When femoral offset is decreased, the abductor muscle origin and insertion remained unchanged. As the greater trochanter underwent medial transfer to the femoral head center, the event must be accompanied by a decrease of the moment arm of the abductors (Fig 3). Abductor muscle force has a known point of application estimated from the muscle origin and insertion on a roentgenogram, but its magnitude is unknown. Accurate measurement of the moment arm of the abductors has fundamental limitations with inherent difficulties [28] because several muscles are involved in the action of hip abduction [23]. Planar radiographs do not characterize the three-dimensional pathway of the musculoskeletal geometry and thus do not allow accurate measurements [28]. With the offset and simultaneous decrease in the moment arm of the abductor, the abductor muscles should increase their force to balance the moment of gravitational force, with increasing abduction force. This change may result in greater tension and contracture of the abductors. Consequently, the operated hip becomes lower, the contralateral (normal) hip becomes higher, and the pelvis is accordingly inclined toward the operated hip, which may result in the formation of type IC PO.

The moment arms of gravitational force and the abductor muscles can be manipulated during reconstructive hip surgery. Similarly, increasing the femoral offset or lateralizing the greater trochanter during total hip arthroplasty increases the mechanical advantage of the abductor musculature by increasing the abductor moment arm, which correlates with the decrease in the joint reaction force [28].

This finding can partly explain the genetic mechanism of type IC PO; its inclination was mainly caused by the contracture of the ipsilateral abductor (gluteus) and tensor fasciae latae [29,30]. However, generation of the mechanism underlying type II PO remains unknown. The contracture of the ipsilateral adductor (such as the iliopsoas) may be the main cause, because PO is a common secondary effect of abduction or adduction hip contracture [7, 8].

Based on these observations, type I PO (especially type IC PO) can be considered irreversible and fixed. If greater tension and contracture of the abductor were present, the adoption of a femoral prosthesis with a normal offset would be difficult intraoperatively. This situation may lead to the shorter offset of the operated hip, which would, in turn, result in the formation of type IC PO. The adequate release of the contracture of the abductor helps to restore the femoral offset intraoperatively, whereas restoration of the normal femoral offset can increase hip stability by preventing impingement and improving soft tissue tension [26], formatting the type IC PO, and maintaining postoperative pelvic balance in THA.

The main limitation of this study was the absence of follow-up. The monocentric nature of the data also presents the possibility of selection bias. More precisely designed studies that involve the anatomy, biomechanics, and finite element analysis of the hip and pelvis are necessary to determine the mechanism contributing to PO.

In summary, PO exhibited no effect on LLI in patients with THA. The results demonstrated that type IC PO was a relatively fixed PO, and type IIC PO was easy to be corrected intraoperatively. Shorter offsets may be caused by the greater tension of the abductor in the operated hip, which may result in the formation of type IC PO. Releasing the abductor’s contracture and restoring femoral offset are important for increasing hip stability and maintaining postoperative pelvic balance following THA.

## Supporting Information

S1 Text The measurement based on the preop-PO. (XLS)

S2 Text The measurement based on the postop-PO. (XLS)
